# Donepezil Rescues Spatial Learning and Memory Deficits following Traumatic Brain Injury Independent of Its Effects on Neurogenesis

**DOI:** 10.1371/journal.pone.0118793

**Published:** 2015-02-25

**Authors:** Tzong-Shiue Yu, Ahleum Kim, Steven G. Kernie

**Affiliations:** Departments of Pediatrics and Pathology & Cell Biology, Columbia University College of Physicians and Surgeons, 3959 Broadway, CHN 10-24, New York, NY 10032, United States of America; Univ. Kentucky, UNITED STATES

## Abstract

Traumatic brain injury (TBI) is ubiquitous and effective treatments for it remain supportive largely due to uncertainty over how endogenous repair occurs. Recently, we demonstrated that hippocampal injury-induced neurogenesis is one mechanism underlying endogenous repair following TBI. Donepezil is associated with increased hippocampal neurogenesis and has long been known to improve certain aspects of cognition following many types of brain injury through unknown mechanisms. By coupling donepezil therapy with temporally regulated ablation of injury-induced neurogenesis using nestin-HSV transgenic mice, we investigated whether the pro-cognitive effects of donepezil following injury might occur through increasing neurogenesis. We demonstrate that donepezil itself enhances neurogenesis and improves cognitive function following TBI, even when injury-induced neurogenesis was inhibited. This suggests that the therapeutic effects of donepezil in TBI occur separately from its effects on neurogenesis.

## Introduction

Traumatic brain injury is a ubiquitous and devastating condition that affects millions of people annually and can be acquired by anyone regardless of genetic background or socioeconomic class. Despite its pervasiveness, the only known effective treatments are supportive and rely heavily on rest and avoidance of recurrent injury. There are still no clear means of improving outcome following traumatic brain injury, though it has been studied extensively for decades. Part of the problem is that this kind of global injury affects many different cell types and how particular subclasses of neurons and glia respond to the same insult may be quite different. In the last decade, injury-induced neurogenesis has fueled tremendous speculation regarding the potential of self-repair after brain injury in general and TBI in particular. After first describing the phenomenon of TBI-induced neurogenesis, we subsequently demonstrated that early progenitors become activated by injury while late-stage progenitors are vulnerable and die [1,2]. More recently, we have shown that genetically controlled ablation of the progenitor pool impairs functional outcome after TBI, demonstrating an adaptive role for injury-induced neurogenesis [3].

In this study, we investigated if the anticholinesterase inhibitor donepezil augmented neurogenesis after injury to further improve recovery in cognition. Anticholinesterase inhibitors are a mainstay of treatment for some neurodegenerative conditions such as Alzheimer disease and have shown promise in treating those who have suffered traumatic brain injuries [4]. Donepezil (Aricept) is the most common clinically used anticholinesterase inhibitor for both TBI and Alzheimer disease, though the exact mechanisms underlying cognitive enhancement are unknown. Recent data suggest that donepezil’s pro-cognitive effects might be due to increasing hippocampal neurogenesis [5–7].

Donepezil is an FDA-approved drug used to treat Alzheimer disease that is minimally toxic, crosses the blood brain barrier, and specifically augments hippocampal neurogenesis. Donepezil’s effects on the brain are, however, ubiquitous and it has been impossible to attribute its pro-cognitive effects to any particular mechanism given these pleiotropic actions. Here, we administered donepezil to nestin-HSV-TK transgenic mice, which allow for temporally regulated ablation of neurogenesis by administration of valganciclovir to establish whether donepezil-dependent hippocampal neurogenesis is beneficial following TBI. In this manner, we specifically investigated normal injury-induced conditions, injury-induced conditions in which neurogenesis is inhibited, injury-induced conditions with administration of donepezil, and injury-induced conditions with administration of donepezil and neurogenesis inhibited. We found that the pro-cognitive effects of donepezil observed following TBI are independent from its effect on injury-induced neurogenesis.

## Materials and Methods

### Animals

Experimental animals were housed and cared for in the Animal Facility at Columbia University Medical Center (CUMC), which is certified by the Association for Assessment and Accreditation of Laboratory Animal Care. All animal experiments were conducted with approval of the Institutional Animal Care and Use Committee at CUMC for the humane and compassionate use of animals in biomedical research. We used transgenic mice, nestin-HSV-TK, which were previously generated on a C57Bl/6 genetic background with no apparent phenotype in the absence of ganciclovir [2]. The gender and total number of nestin-HSV-TK mice used in each experimental groups were described as follows: sham mice treated with vehicle and normal chow (5 males and 4 females), sham mice with donepezil and normal chow (6 males and 5 females), injured mice with vehicle and normal chow (9 males and 5 females), injured mice with donepezil and normal chow (6 males and 5 females), sham mice with vehicle and valganciclovir chow (7 males and 3 females), sham mice with donepezil and valganciclovir chow (8 males and 4 females), injured mice with vehicle and valganciclovir chow (5 males and 6 females), injured mice with donepezil and valganciclovir chow (5 males and 5 females). All animals were between 8 and 9 weeks of age when injured or mock-injured.

### Controlled cortical impact injury

To perform controlled cortical impact (CCI), a standard protocol using a controlled cortical impact device (Leica Impact One, Leica Biosystems) was used to generate brain injuries as described previously [3]. Eight-week-old nestin-HSV-TK mice were anesthetized with isoflurane and then placed in a stereotactic frame. A midline incision was made, the soft tissues were reflected, and a 5x5 mm craniotomy was made between bregma and lambda and 1 mm lateral to the midline. The injury was generated with a 3 mm stainless steel tipped impact device with a deformation of 0.7 mm, constant speed of 4.4 m/s and duration of 300 milliseconds. After surgery, the scalp was fastened with sutures and the mice were allowed to recover. For sham operations, mice received all the procedures noted above but the impact was not performed. After surgery, all mice were received 5-Chloro-2-deoxyuridine (CldU) (88.5mg/Kg; Sigma) 3 days after injury for 3 consecutive days.

### Donepezil, CldU, valganciclovir administrations

Donepezil (2mg/Kg, Ivy Fine Chemicals) was injected intraperitoneally once a day for five consecutive days (M-F) for two weeks starting from post-operation day 3. CldU (88.5mg/Kg, Sigma) was delivered via intraperitoneal injection once a day for 3 days following donepezil injection. Valganciclovir (Valcyte, Genetech) was mixed with regular grain-based rodent diet to produce 900mg/Kg valganciclovir chow (Custom Animal Diets, LLC, Easton, PA). Valganciclovir chow was provided *ad libitum* for ganciclovir-treated groups during the experimental periods.

### Morris water maze

The Morris water maze was conducted by an experimenter blinded to the experimental treatment conditions as previously described [3]. Briefly, a white circular pool was filled with water that was mixed with gothic white, nontoxic paint to make it opaque. A circular platform (15 cm in diameter) was submerged 1 cm beneath the surface of the water. A number of extra-maze cues surrounded the pool in the testing room. Training in the Morris water maze was conducted for 11 consecutive days, and each training day consisted of 3 individual trials with an inter-trial interval of 1 minute. During training trials, mice were allowed to swim freely until they found the hidden platform, and if they did not find the platform in 60 seconds, they were guided to the platform by the experimenter. For each training day, data were averaged across the three trials. A probe trail was performed on day 12. The hidden platform was removed, and the mice were placed in the pool and allowed to swim for 60 seconds. All data were collected using Ethovision software (Noduls, version 9).

### Immunohistochemistry

All mice were deeply anesthetized with isoflurane before perfusion. Transcardiac perfusion was performed with phosphate-buffered saline (PBS), followed by 4% paraformaldehyde (PFA) in 1xPBS. Following post-fixation in PFA/1xPBS overnight, brains were dissected and embedded in 3% agarose. Serial 50μm sections were cut with a vibratome (VT1000S, Leica). All sections encompassing the hippocampus were collected sequentially in 6-well plates. Free-floating sections were used for immunohistochemistry. For CldU staining, all sections from a single well were washed with PBS and rinsed with water. Sections were denatured with 0.1N HCl and then washed with 0.3% Tritox X-100/1xPBS (wash buffer) 3 times and blocked with 5% normal donkey serum (Jackson ImmunoResearch Labs) with buffer for 1 hour. 1:500 rat anti-BrdU antibody (OBT0030, Ab Serotec) and 1:500 mouse anti-NeuN antibody (clone A60, E0003, Fisher Scientific) was used to label CldU overnight. The following day, sections were incubated with Alexafluoro 594 donkey anti-mouse and Alexafluoro 647 donkey anti-rat IgG (all 1:200, Jackson ImmunoResearch) for 3 hours. The sections were placed on slides and coverslipped.

### Quantification and microscopy

CldU-labeled cells in the dentate gyrus were counted using an unbiased stereological approach. A one-in-six series of sections covering the entire hippocampus in its rostrocaudal extension was cut with a vibratome (Leica) at 50μm, immunostained, and visualized under light microscopy with the peroxidase/diaminobenzidine method. Cells were counted using an optical fractionator and stereological image analysis software (StereoInvestigator; MicroBrightField) operating a computer-driven microscope (Imager M2, Zeiss) regulated in the *x*-, *y*-, and *z*-axes. Areas to be counted were traced with a 20x objective lens, and sample frames (70 X 70μm) were selected at random by the image analysis software. Cells in the counting frame (40 X 40μm) were counted under a 63X oil objective lens. To avoid oversampling, the uppermost and lowermost focal planes were excluded and the Gundersen coefficient of error was less than 0.1.

For cell quantification of NeuN/CldU-labeled cells, we used a validated method to account for oversampling as described in detail previously [2,3,8]. A Zeiss Imager M2 microscopy was used to determine colocalization of two immunohistochemical markers and cells were quantified with a Carl Zeiss 40X objective lens. Cell quantification was performed on the ipsilateral and contralateral sides of the dentate gyrus in all groups. Sections were processed and collected as above and picked randomly and stained with antibodies as free-floating sections to allow for equal antibody penetration between sections. Sections were mounted using immunomount (Vectashild D, Vector Labs). Antibody penetration and signal intensity were found to be evenly distributed throughout the entire 50μm section and was verified on multiple sections from different animals. Each blade of the dentate gyrus was counted separately at 40X with 1.5X optical zoom magnification. Blinded quantification was performed on cells with fully colocalized antibodies in the granular layer of the dentate gyrus. Every NeuN/CldU- and CldU-labeled cell was counted to get the percentage of total CldU-labeled cells co-labeled with NeuN (NeuN/CldU-labeled newborn neurons) to calculate the estimated number of newborn neurons.

Feeding nestin-HSV-TK mice with valganciclovir chow resulted in ablation of the majority of dividing neural progenitors, and rare CldU-labeled cells were observed. Therefore, we were unable to use unbiased stereology to quantify CldU-labeled cells in valganciclovir chow-fed nestin-HSV-TK mice. To determine whether valganciclovir was efficient in ablating dividing neural progenitors in nestin-HSV-TK mice all CldU-labeled cells were counted. A Zeiss Imager M2 microscopy was used to determine number of CldU-labeled cells with a Carl Zeiss 40X objective lens. Cell quantification was performed on the ipsilateral and contralateral dentate gyrus in all groups. Sections were processed and collected as described as aboved. Each blade of the dentate gyrus was counted separately at 40X with 1.5X optical zoom magnification. Blinded quantification was performed on cells in the granular layer of the dentate gyrus. Every CldU-labeled cell was counted manually in a blinded manner and raw data without estimation or adjustment were presented for statistical analysis.

### Statistical Analysis

Statistical analysis was performed with Prism 6 software. All behavioral data were analyzed using two-way ANOVA with Fisher’s LSD *post-hoc* analysis unless otherwise specified. All non-behavioral data were analyzed using one-way ANOVA with Fisher’s LSD *post-hoc* analysis or unpaired Student’s *t* test.

## Results

### Donepezil treatment enhances neurogenesis

**Fig 1:**
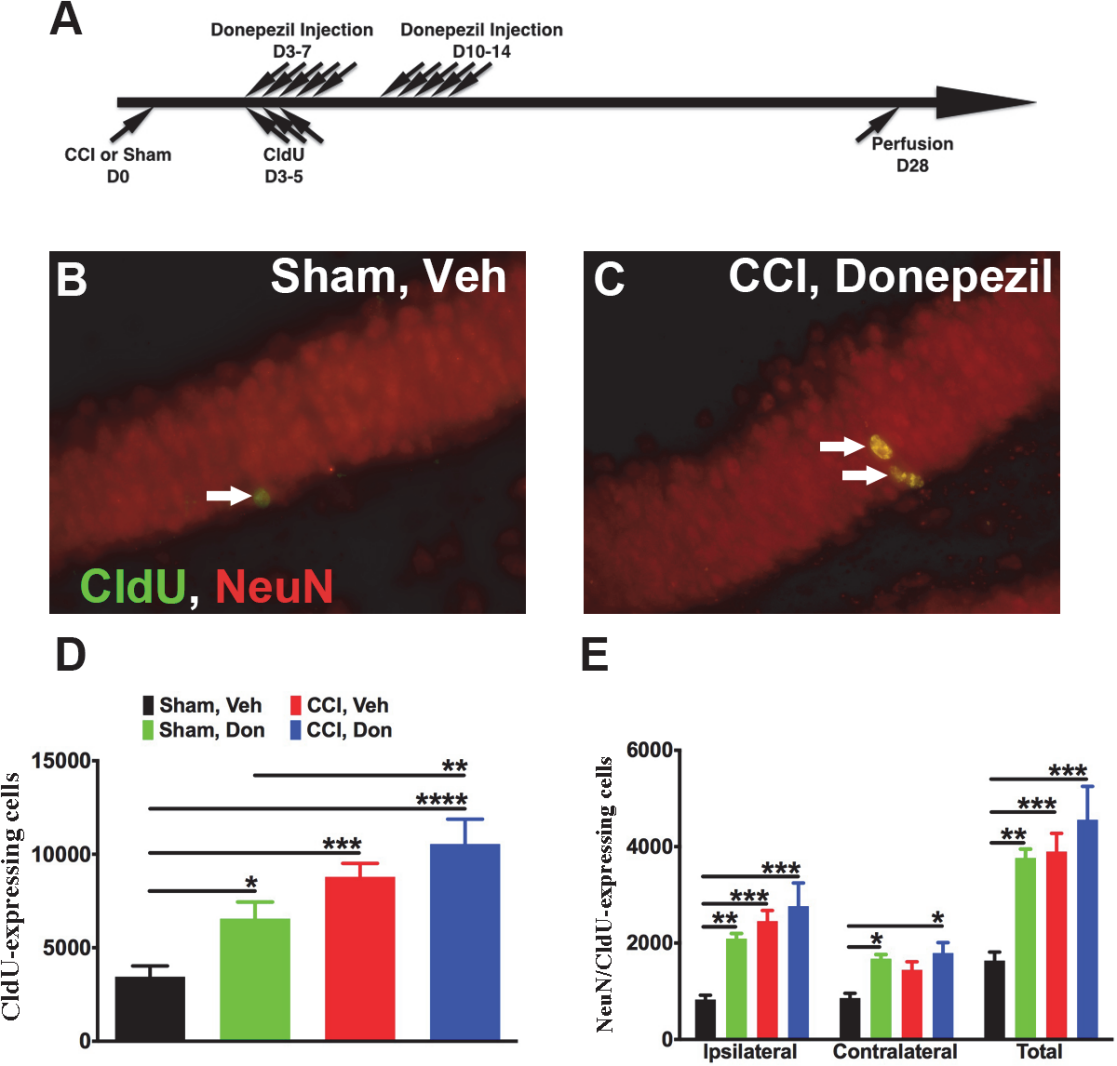
Donepezil treatment enhanced adult neurogenesis in the dentate gyrus in sham-operated but not injured mice. (A) Surgery was performed on 8-week old mice (D0). Starting from D3, vehicle or donepezil was given for 5 days a week, for 2 weeks. CldU was also injected for 3 days to label the dividing cells. Mice were perfused for further analysis 4 weeks after surgery. (B, C) Representative pictures show that more newly born neurons (arrows) were observed in CCI-operated mice after being treated with donepezil when compared to control. (D) Unbiased stereology quantification revealed that donepezil treatment resulted in a significant increase in CldU-labeled cells in the dentate gyrus in sham-operated but not injured mice. (E) Treatment with donepezil led to a significant increase in number of newborn neurons in sham-operated mice. One-way ANOVA with Fisher’s multiple comparisons post-hoc analysis in D and E with * *p*<0.05, ** *p*<0.01, *** *p*<0.001, **** *p*<0.0001. Sham: *sham-injured*, CCI: *controlled cortical injury*, Veh: *vehicle*, Don: *donepezil*, Ipsi: *ipsilateral*, Contra: *contralateral*. Number of mice used was 9 (Sham, Veh), 6 (Sham, Don) 6 (Sham, Don), 5 (CCI, Veh), 5 (CCI, Don), Scale bar in C is 50μm.

We first sought to determine whether donepezil enhances hippocampal neurogenesis in control and injured animals. Mice with sham or CCI injury received one injection of donepezil (2 mg/Kg) daily for five days (M-F) for two consecutive weeks starting three days after injury. Previous work by us demonstrated that proliferation of neural progenitors in the injured mice peaks 3 days after injury and donepezil has been shown in many studies to enhance neurogenesis in mice. Therefore, mice received CldU (85.55 mg/Kg) once a day starting three days after surgery for 3 consecutive days to determine if donepezil affected the peak proliferation of injury-induced neurogenesis (Fig. 1A). Mice were sacrificed four weeks later for immunostaining to quantify numbers of CldU-labeled cells and newborn neurons. Unbiased stereology was used to quantify numbers of CldU-labeled cells in the dentate gyrus. Donepezil treatment resulted in a significant increase in CldU-labeled cells in sham-operated mice but not injured mice (Fig. 1D). Moreover, the number of newly born neurons (CldU/NeuN) was increased significantly in sham-operated mice after donepezil treatment (Fig. 1B, C and E). In injured brains, donepezil treatment did not increase neurogenesis on the injured side when compared with vehicle-treated controls, though it did increase neurogenesis on the contralateral side of injured mice (Fig. 1E). Therefore, donepezil treatment resulted in a highly significant increase of newly born neurons in sham-injured mice and on the contralateral side of injured mice.

### Donepezil treatment improved performance in spatial memory

**Fig 2:**
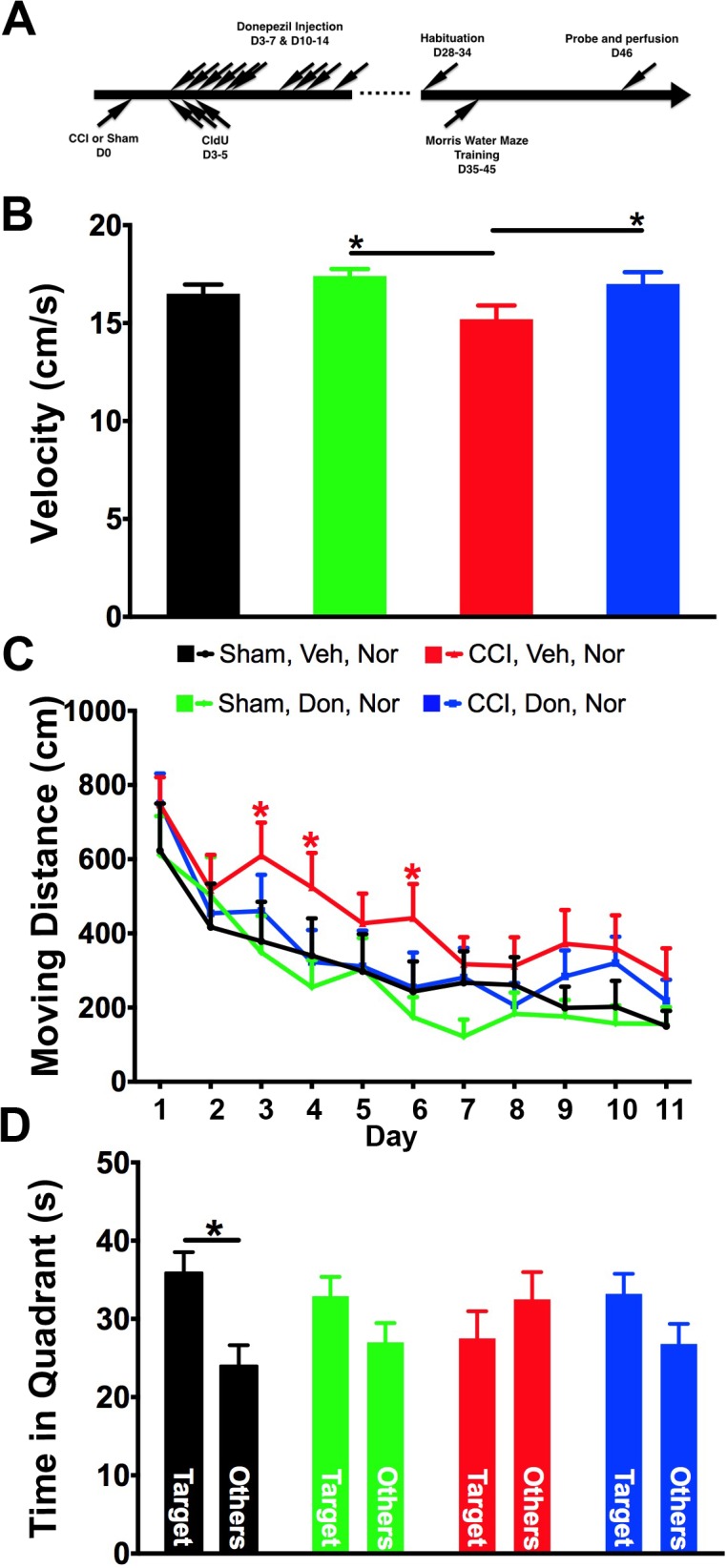
Donepezil treatment improved spatial learning and memory in injured mice. (A) As depicted in the scheme, sham- and injured mice received either vehicle or donepezil for two weeks. Four weeks after surgery, mice were habituated for the Morris water maze. Mice were trained to find the platform for 11 consecutive days and a probe test was performed at day 12. (B) Swimming speed of injured mice was slower than the other groups if no treatment was given. Donepezil treatment reversed the motor deficit in swimming. (C) Because of deficit in swimming speed, the moving distance to finding the platform was used as an indicator of learning ability of a novel task. Consistent with our previous study, injured mice without treatment took the longest distance in finding the platform. However, injured mice took shorter paths after donepezil was given. (D) Time mice spent in the target quadrant versus the summation in all other quadrants was used as an indication of memory ability. Mice in all groups remembered the location of the platform, however, injured mice without any treatment spent less time in the target quadrant while the rest spent more time in the target area. Two-way ANOVA with Fisher’s LSD multiple comparisons test was used in B and C. One-way ANOVA with Fisher’s LSD multiple comparisons was used in D. * *p*<0.05, Sham: *sham-injured*, CCI: *controlled cortical injury*, Veh: *vehicle*, Don: *donepezil*. Number of mice used was 9 (Sham, Veh), 11 (Sham, Don) 14 (CCI, Veh), 11 (CCI, Don).

By using CldU-labeling, we demonstrated increased numbers of neurons in the dentate gyrus in donepezil-treated mice, in both injured and sham-injured controls. To investigate whether treatment improved learning ability and memory, the hidden platform version of the Morris water maze was performed (Fig. 2A). Mice were trained to find the platform according to the visible cues surrounding the water tank for 3 trials per day, on 11 consecutive days, and the moving distance finding the platform represented their ability in learning to find the platform. As suggested by swimming speed, the motor activity in injured mice that received no treatment was impaired (Fig. 2B), therefore, the moving distance was used to determine the learning ability of the mice. Consistent with our previous observations, the moving distance of injured mice without any treatment was significantly increased for finding the position of the hidden platform, especially during early periods of training (Fig. 2C). Interestingly, injured mice treated with donepezil learned to find the location of the hidden platform similar to sham-operated mice (Fig. 2C). Therefore, donepezil treatment improved the recovery of injured mice in spatial learning ability to that of sham-injured controls.

On day 12, the platform was removed to perform the probe test and determine if mice remembered the position of the platform. Time spent in the target quadrant was compared with times in the other three quadrants to indicate how well the memory is preserved. As expected, sham-operated mice, both with and without donepezil, spent most of the time in the target zone. However, injured mice spent relatively less time in the target zone compared to all other groups (Fig. 2D). Although there was no significant difference in time spent in the target quadrant zone between injured mice treated with vehicle or donepezil, injured mice spent more time in the target zone in a manner similar to what was observed in controls after donepezil treatment (Fig. 2D). Therefore, donepezil improves learning and spatial memory in mice after injury.

### Valganciclovir treatment inhibits neurogenesis in nestin-HSV-TK mice

**Fig 3:**
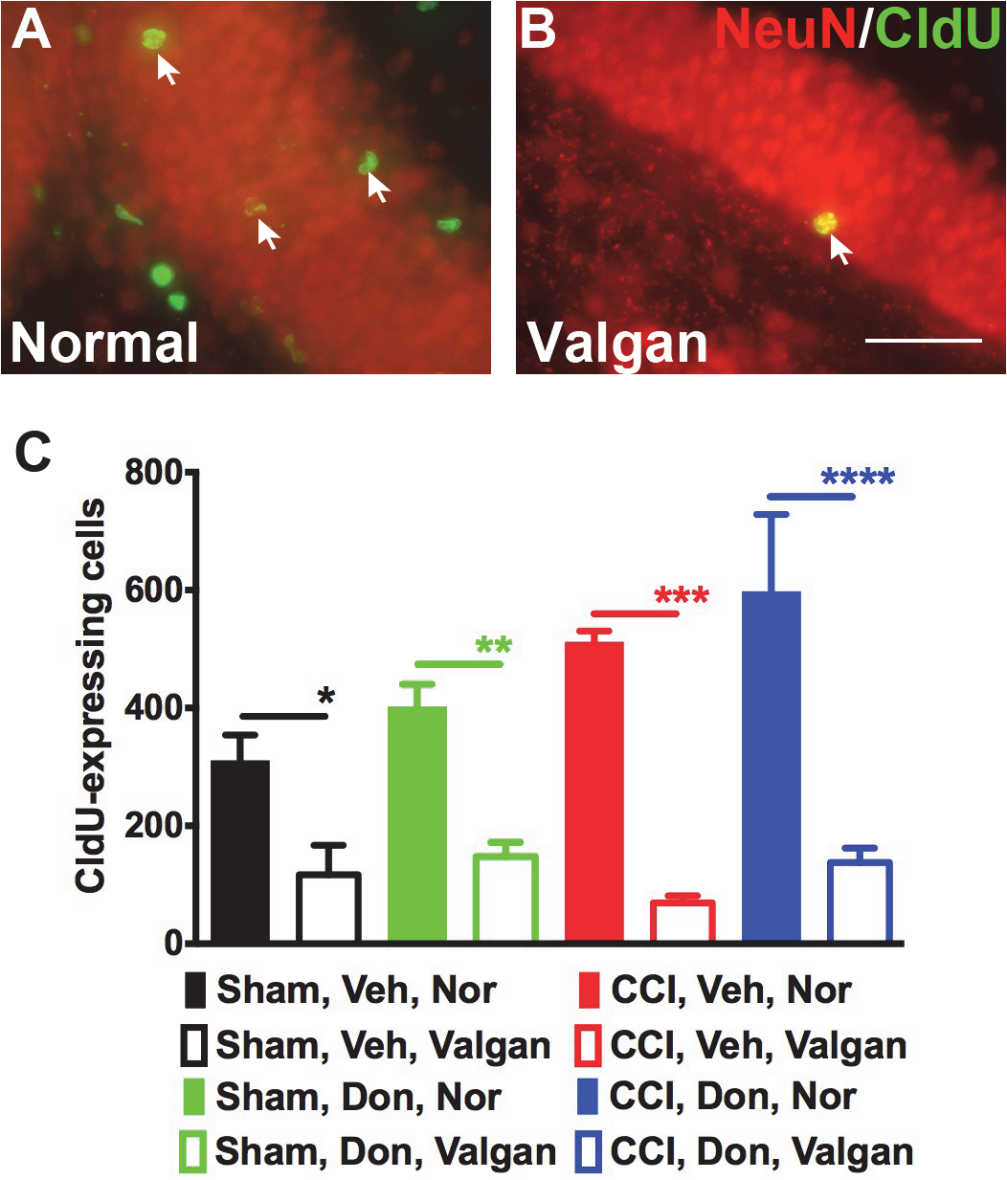
Valganciclovir chow inhibited neurogenesis in nestin-HSV-TK mice. (A and B) The representative section demonstrates that rare CldU/NeuN double-labeled cells (arrow) were observed in the dentate gyrus after mice were fed with valganciclovir chow for 4 consecutive weeks (40x). (C) Quantification of CldU-labeled cells in the dentate gyrus of nestin-HSV-TK mice demonstrated that neurogenesis was efficiently inhibited in all the experimental groups. One-way ANOVA with Fisher’s LSD multiple comparisons was used in C, * *p*<0.05, ** *p*<0.01, ***p<0.001, **** *p*<0.0001. Sham: *sham-injured*, CCI: *controlled cortical injury*, Veh: *vehicle*, Don: *donepezil*. Nor: *normal chow*, Valgan: *valganciclovir*. Number of mice used was 4 (Sham, Veh, Nor), 5 (Sham, Veh, Valgan) 6 (Sham, Don, Nor), 5 (Sham, Don, Valgan), 3 (CCI, Veh, Nor), 3 (CCI, Veh, Valgan), 4 (CCI, Don, Nor), and 5 (CCI, Don, Valgan). Scale bar in B is 50μm.

As demonstrated in Fig. 1, donepezil treatment did not alter the fate of injury-induced neurogenesis and was demonstrated to potently increase neurogenesis. In addition, for injured animals, treatment with donepezil increases neurogenesis on the contralateral side, which may play a role in its ability to improve recovery following TBI. We previously demonstrated that nestin-HSV-TK mice that we generated can be used to inducibly ablate neurogenesis following injury [3]. Since donepezil, an acetylcholine esterase inhibitor, acts on cholinergic neurons and enhances neurogenesis, we took advantage of nestin-HSV-TK transgenic mice to inhibit neurogenesis and investigate whether donepezil-mediated improvement in spatial learning ability and memory was due to enhancing neurogenesis. The nestin-HSV-TK transgenic mice were fed with normal or valganciclovir chow starting immediately after surgery for four weeks to inhibit neurogenesis (Fig. 3A and B). After feeding mice valganciclovir for four weeks, CldU-labeled cells in the dentate gyrus were rarely found in each experimental group compared to the ones fed normal chow (Fig. 3C). Hence, neurogenesis induced by donepezil injections and CCI was inhibited efficiently by administering valganciclovir to nestin-HSV-TK mice.

The pro-cognitive effects of donepezil occur independently from injury-induced neurogenesis.

**Fig 4:**
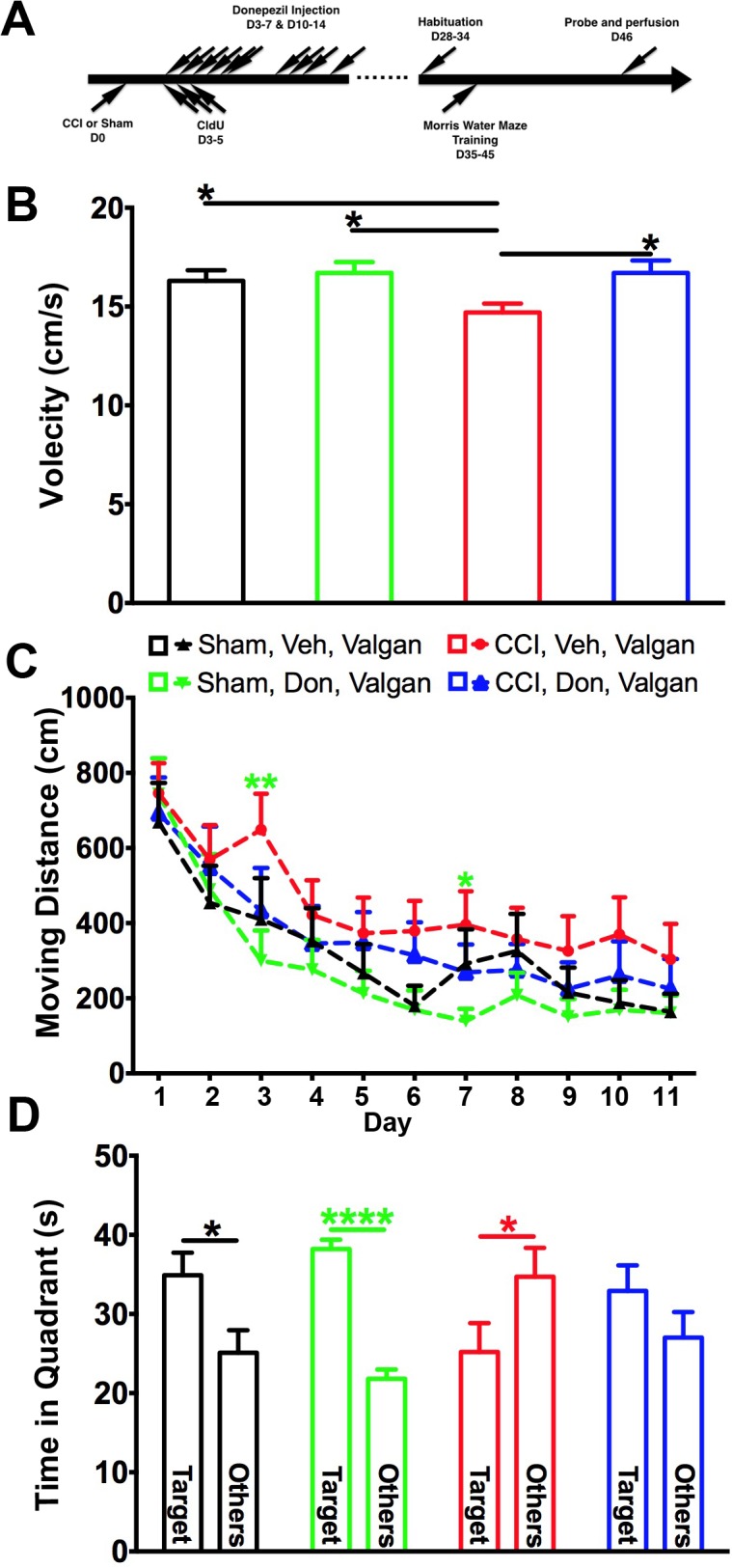
The effect of donepezil in spatial learning and memory in injured mice is independent from neurogenesis. (A) As depicted in the scheme, sham- and injured mice received either vehicle or donepezil for two weeks. Four weeks after surgery, mice were habituated for the Morris water maze. Mice were trained to find the platform for 11 consecutive days and a probe test was performed at day 12 Mice were fed with valganciclovir chow immediately after surgery until the end of all experiments. (B) Swimming speed in injured mice without neurogenesis and treatment was slower than controls. Donepezil treatment was able to overcome the impairment even in the absence of neurogenesis. (C) Consistent with previous observations, in the absence of neurogenesis the injured mice took the longest path to locate the hidden platform if no treatment was given. However, the performance improved after injured mice were treated with donepezil even while neurogenesis was inhibited. (D) Injured mice without neurogenesis and donepezil treatment spent significantly less time in the target zone when compared with the summation of time spent in other zones (open blue bars). However, treatment with donepezil reversed this deficit (open red bars). Two-way ANOVA with Fisher’s LSD post-hoc analysis was used in A and B. One-way ANOVA with Fisher’s LSD analysis was used in C. * *p*<0.05, **** *p*<0.001. Sham: *sham-injured*, CCI: *controlled cortical injury*, Veh: *vehicle*, Don: *donepezil*. Nor: *normal chow*, Valgan: *valganciclovir*. Number of mice used was 10 (Sham, Veh, Valgan), 12 (Sham, Don, Valgan), 11 (CCI, Veh, Valgan), and 10 (CCI, Don, Valgan).

We efficiently inhibited neurogenesis in donepezil-treated nestin-HSV-TK mice given valganciclovir chow immediately after surgery until behavioral experiments were finished (Fig. 4A). The hidden platform version of the Morris water maze was next performed to reveal the role of inhibiting neurogenesis in spatial learning ability and memory in donepezil-treated and injured animals. Again, the velocity was slower in the injured mice that were fed with valganciclovir chow compared to other groups (Fig. 4B). However, the swimming speed was not comparable to controls after donepezil was given (Fig. 4B). Following treatment with donepezil, sham-operated mice performed similarly in learning to locate the platform whether neurogenesis was intact or not (Fig. 4C). Consistent with our previous study, injured mice without neurogenesis were impaired in their ability to find the hidden platform when compared to the other three groups (Fig. 4C). However, when injured mice that also had neurogenesis ablated were treated with donepezil, they performed in a comparable manner to sham-injured mice (Fig. 4C).

Inhibition of neurogenesis in sham-operated mice did not affect the time that mice spent in the target quadrant during the probe test regardless of donepezil treatment (Fig. 4D). As expected, vehicle-treated injured mice with inhibition of neurogenesis spent significantly less time in the target quadrant. However, donepezil treatment reversed this deficit (Fig. 4D). Therefore, although donepezil is a potent inducer of hippocampal neurogenesis, its pro-cognitive effects seen following injury appear to occur independently of these neurogenic properties.

## Discussion

In this study, we investigated whether donepezil, an FDA-approved drug used to treat Alzheimer disease, contributes to pro-cognitive recovery in a neurogenic-specific way following traumatic brain injury. By quantifying dividing cells labeled by CldU in the dentate gyrus, a significant increase of newborn neurons was observed in sham-operated mice treated with donepezil, a result that was also seen following injury on the contralateral side. To further test the effect of donepezil in cognition, the hidden platform version of the Morris water maze was performed. Consistent with previous studies, the moving distance was longer in injured vehicle-treated mice for locating the platform with the surrounding visible cues during the training period. Although injured mice ultimately learned the task, they did not remember as well as other groups as revealed in the probe test. Interestingly, the deficits in learning and memory were rescued by treatment with donepezil. Furthermore, these rescued deficits were maintained even when neurogenesis was inhibited using previously validated nestin-HSV-TK mice. Therefore, it appears that donepezil improves recovery in spatial learning and memory in injured mice through a neurogenesis-independent mechanism.

Donepezil is an acetylcholinesterase inhibitor that extends the effect of acetylcholine on neurons. Although several studies suggest that donepezil enhances neurogenesis in adult rodents and improves cognition in aged rats [5–7,9,10], the mechanisms underlying these observations remain unknown. Consistent with previous studies, a significant increase in newborn neurons was noted in donepezil-treated sham-operated mice and the level of enhancement in neurogenesis was close to that observed in injured mice. Enhanced neurogenesis is known to improve the function of several hippocampal-based behavioral tasks [7,11–13]. Here, we observed that donepezil rescued the effects of inhibiting neurogenesis following TBI. Furthermore, inhibition of neurogenesis in injured mice did not affect the pro-cognitive effects of donepezil.

Several studies have concluded that CCI affects motor function as indicated in swimming speed [14–17] and here, donepezil treatment resulted in a significant increase in swimming speed in injured mice (Fig. 2D). This suggests that one of the beneficial effects of donepezil on injured mice might be through improvement in motor activity. A recent study using donepezil to treat rats after CCI described an opposite observation [18]. However, in that study, motor function was tested with beam balance and beam walk five days after injury. No motor tasks were performed at similar time points as the present study, so it remains unknown if similar improvement in motor activity might be observed over time in injured rats with donepezil treatment. In the present study, we presented the learning data from the Morris water maze as moving distance rather than latency time in order to control for differences in swimming speed and therefore demonstrate that its effects on learning and memory are independent of its effects on motor function.

The lack of more obvious performance differences in the Morris water maze in our study might be due to overtraining. Mice were trained for three trials a day on eleven consecutive days intentionally to emphasize deficits in learning in injured mice. However, this might contribute to a lack of difference in performance between vehicle- and donepezil-treated sham-operated mice. Previous work demonstrated that administration of donepezil for four weeks in mice resulted in an increase in neurogenesis and insulin-like growth factor 1 (IGF-1) in the hippocampus and improved spatial learning and memory [7]. It was not clear whether the improvement of cognitive function was from an increase of IGF-1, neurogenesis, or both in that study. Two-week treatment with donepezil in sham-operated mice in our study was sufficient to enhance neurogenesis, but not beneficial in improving spatial learning and memory revealed by the Morris water maze. It would be interesting to test if two-week treatment with donepezil induces IGF-1 in the hippocampus since IGF-1 has been suggested as a beneficial factor in improving cognitive function [19,20].

As expected, injured mice suffered deficits in learning a new skill, which persisted as impaired memory when learned without any treatment with donepezil. Interestingly, treatment with donepezil improved the performance of injured animals to the level of sham-operated controls. Although it is an acetylcholinesterase inhibitor that strengthens the activities of the cholinergic system, it is not yet known how cholinergic neurons contribute to recovery from TBI. One possible mechanism investigated in this study is injury-induced neurogenesis. The lack of neurogenesis in nestin-HSV-TK transgenic mice did not result in deficits in spatial learning and memory in donepezil-treated injured mice. This suggests that the mechanism underlying donepezil-mediated recovery in spatial learning and memory in injured mice is not through hippocampal neurogenesis.

Although donepezil induced a significant increase in neurogenesis in sham-injured mice, this effect was more modest in donepezil-treated injured mice. It is possible that injury activated all available neural progenitors to develop into newborn neurons as indicated in our previous study [2], and that this occurs via a similar mechanism by which donepezil enhances neurogenesis. In addition, although the mechanisms may be totally unrelated, the degree to which existing progenitors can be stimulated might be limited by whether this occurs secondary to injury or pharmacologic stimulus. If this is the case, it suggests that donepezil is not able to activate the quiescent type I neural stem cells.

Unlike what was seen in injured mice, there was no significant effect observed in learning and memory ability in the water maze task in sham-operated mice treated with donepezil. However, donepezil-treated sham-operated mice without neurogenesis performed better in the probe task compared to the comparative control group with neurogenesis. Although a recent study demonstrated that neurogenesis interferes with existing remote memory [21], it does not provide an explanation for our observations since neurogenesis was inhibited before the beginning of the training and probe tasks. Overall, these data suggest that in sham-operated mice the donepezil-induced neurogenesis might interfere with newly formed memories, although further study is required to confirm this observation.

This study demonstrates that donepezil improves recovery of spatial learning and memory in injured mice and that this effect occurs largely independent from injury-induced neurogenesis. This suggests that enhancing cognition following TBI occurs through new neuron-dependent and independent mechanisms and provides a potential route for improving cognitive recovery in brain-injured patients.
